# Annotations

**Published:** 1898-01-29

**Authors:** 


### ANNOTATIONS.

### The Adelaide Hospital.

THE Government at Adelaide seems to be in difficulties again. As our readers will remember, in consequence of the Government being unable to satisfactorily replace the members of the medical and surgical staff who had resigned, the Government brought out from England Dr. Leith Napier and Dr. Ramsay Smith, whom they installed in their places. After a time the junior resident staff also resigned, and the Government brought out others to do their work, among them Dr. Morrison. By degrees it would appear that an honorary staff is being got together again, notwithstanding the fulminations of the British Medical Association, and on the new members of the honorary staff taking office it has become necessary to take away some of the authority which seems to have drifted into the hands more especially of Dr. Morrison. On this he has threatened to resign, and the whole affair has been brought before the Legislative Council! Dr. Morrison asserts that his contract with the Agent-General was to go out to Adelaide as assistant physician, and he now refuses to do house-physician's work under the physicians, while the governors maintain that he went out to take up the work of the junior staff who had resigned, and whose duty it certainly was to do this work. What will happen it is impossible to say, because the whole thing, being under Government management, becomes political; but it is quite clear that in England no voluntary hospital would allow the continuance for a single day of such a condition of discord as is displayed in the correspondence between the officials involved. The renewed attention now drawn to the Adelaide Hospital must make members of the British Medical Association wonder what their next move in the matter will have to be. Are all who join the staff to be expelled?

### The "British Medical Journal."

THE British Medical Association is much to be congratulated upon the common-sense action of its Council in unanimously handing over the editing of its *Journal* to those to whom in late years its continued success has been largely due. At the meeting of the Council held on January 19th, Dr. Dawson Williams, the Assistant Editor, was appointed Editor, and Mr. C. Louis Taylor, who has been Sub-Editor for the last eleven years, was appointed Assistant Editor. Those who have watched the progress of the *British Medical Journal* during recent years, and have known the part taken by Dr. Dawson Williams and Mr. Louis Taylor in maintaining its scientific accuracy, its literary style, and its professional status, will best understand how great a disaster it would have been to the Association if any other decision had been arrived at. Mr. Ernest Hart was, before all things, a journalist. But a large part of every issue of the *British Medical Journal* has had but small relation to the sort of journalism in which he revelled, and thus, while Mr. Hart impressed his individuality upon the leaders and the central pages, the organisation and arrangement of the more scientific portions of the paper—the portions which, in the eyes of thousands who care but little for medical politics, really constitute the *Journal*—have been in the hands of those who have been now promoted. For seventeen years Dr. Williams has been connected with the editorial staff, and during late years he has again and again had full editorial responsibility thrown upon him for long periods. But the satisfaction which the members of the British Medical Association will feel at his appointment as editor will not, we fancy, turn entirely, or even mainly, on the fact that he is a trained journalist, greatly as that will conduce to the stability of the paper, but rather on the fact that the editorship of their *Journal* has fallen into the hands of one who is well known as a man of science, a hospital physician, an investigator in the field of pathology, a careful clinical observer and a man who stands well with his professional brethren. In the hands of such an editor they will be content that their journal will be safe both as a professional organ and as a scientific record. So modestly and unobtrusively have the inner workings of the *British Medical Journal* been carried on that many of its readers perhaps hardly know of the existence of Mr. Louis Taylor. This makes it all the more necessary that we should here say how much of the pleasure that the members of the Association derive from the reading of their *Journal* depends on his careful supervision. Few probably are aware of the difficulties of conducting a paper the contributors to which are legion, while the mass of them are only occasional writers. The manufacture of a readable journal from such sources is no easy task, and we have no doubt that many contributors owe much to the encyclopædic knowledge which Mr. Taylor brings to bear upon his work. An extensive acquaintance with many languages, a sort of instinct for spotting false quotations, a sympathetic manner, a kindly readiness to hear both sides of every question, and a faculty withal of seeing the humorous side of each render him not only a charming colleague and companion, but an invaluable acquisition to the staff. Mr. Ernest Hart did many good things for the British Medical Association, but in few things did he do better than in retaining for the *Journal* the services of Dr. Dawson Williams and Mr. Louis Taylor, and we are glad that the Council has seen well to follow in his footsteps.

### The Royal Dundee Infirmary.

THE board of directors of the Dundee Royal Infirmary have decided, in applying for a new charter, to remove a disability which has hung over the honorary medical staff for the past 20 years. When the present charter was obtained in 1877 a clause was inserted which provided that "no person holding any office or emolument in the establishment shall be eligible to be chosen a director," and in accordance with this clause the members of the medical staff who had always had seats on the board not only ceased to be directors, but were rendered ineligible for election as such by their fellow governors. It is now proposed to insert in the new charter a clause to the effect that three members, "but not more," of the honorary medical staff may henceforth be elected to the directorate by the governors. Of course, this merely removes a disability. If they obtain seats they will have to be elected by the governors, and will in no way be representative of their colleagues, who, we suppose, will still continue to have right of access to the meetings of the board (without voting power) as at present.

